# OligoSpawn: a software tool for the design of overgo probes from large unigene datasets

**DOI:** 10.1186/1471-2105-7-7

**Published:** 2006-01-09

**Authors:** Jie Zheng, Jan T Svensson, Kavitha Madishetty, Timothy J Close, Tao Jiang, Stefano Lonardi

**Affiliations:** 1 Department of Computer Science & Engineering, University of California, Riverside, CA 92521, USA; 2 Department of Botany & Plant Sciences, University of California, Riverside, CA 92521, USA

## Abstract

**Background:**

Expressed sequence tag (EST) datasets represent perhaps the largest collection of genetic information. ESTs can be exploited in a variety of biological experiments and analysis. Here we are interested in the design of overlapping oligonucleotide (*overgo*) probes from large *unigene *(EST-contigs) datasets.

**Results:**

OLIGOSPAWN is a suite of software tools that offers two complementary services, namely (1) the selection of "unique" oligos each of which appears in one unigene but does not occur (exactly or approximately) in any other and (2) the selection of "popular" oligos each of which occurs (exactly or approximately) in as many unigenes as possible. In this paper, we describe the functionalities of OLIGOSPAWN and the computational methods it employs, and we report on experimental results for the overgo probes designed with it.

**Conclusion:**

The algorithms we designed are highly efficient and capable of processing unigene datasets of sizes on the order of several tens of Mb in a few hours on a regular PC. The software has been used to design overgo probes employed to screen a barley BAC library (*Hordeum vulgare*). OLIGOSPAWN is freely available at .

## Background

For most organisms, expressed sequence tag (EST) datasets represent the largest collection of genetic sequences available. As of June 2005 more than forty organisms have more than 100,000 ESTs in GenBank dbEST [1], including barley (*Hordeum vulgare*) with over 395,000 ESTs. Most ESTs contain only part of the transcribed sequence of a gene, generally 200–800 bases from one end of a cDNA clone. In order to obtain extended, and in many cases complete, transcript sequences, raw EST data is processed through several steps to produce a "unigene" dataset that represents the full complexity of the initial EST collection. Processing steps include removal of vector and low quality sequences followed by clustering into assemblies, from which consensus sequences are referred to as *unigenes*. In the case of barley, as of February 2005 the collection has over 53,000 unigenes comprising a total of more than 40 megabases. Unigene datasets for numerous organisms can be obtained from GenBank [2], TIGR [3] and various organism-specific sources (e.g., HARVEST [4]).

Given a collection of unigenes, OLIGOSPAWN [5] serves two complementary purposes arising in the selection of oligos for overgo probes. Overgo probes, first described by Ross *et al*. [6,7], are produced using two oligos that are complementary to each other and anneal to form a double-stranded region. First, OLIGOSPAWN can identify short oligos that are *unique *to each unigene in the database. Second, it can select oligos that are *popular *among the unigenes. More precisely, a unique oligo is one that appears in one unigene but does not occur (exactly or approximately) in any other, and a popular oligo is an oligo that occurs (exactly or approximately) in the largest number of unigene sequences.

Typical applications of unique oligos are the design of PCR primers and the selection of probes for microarray studies. For BAC library screening, the purpose of unique oligos is to have the means to unambiguously link each probe to its specific gene-bearing BAC clones [7]. The associated computational problem has been studied quite extensively (see, e.g., [8-10] and references therein), but practical implementations usually are based on the "all-against-all BLAST" strategy and hence very slow on large datasets. Among the primer/probe design softwares, a partial list would contain PRIMER3 [11], OLIGOWIZ [12], OLIGOARRAY [10,13], and PROBEMER [14], SOOP [15], and OVERGO MAKER [16] among others.

In the context of BAC library screening, the purpose of popular oligos is to identify the largest possible list of gene-bearing BAC clones using the smallest possible number of probes (i.e. a "greedy" approach). This strategy is aligned with the desire to economize the identification of what may be only a small portion of gene-bearing fragments from an entire genome. Our interest in popular oligos arises from screening a large collection of BAC clones for barley. It has been shown previously by a number of independent methods that the expressed genes in *Triticeae *are concentrated in a small fraction of the total genome. In barley, this portion of the genome, often referred to as the *gene-space*, has been estimated to be only 12% of the total genome [17]. If this is indeed true, then perhaps only 12%–20% of the clones in a typical BAC library would carry expressed genes, and therefore also the vast majority of barley genes could be sequenced by focusing only on this portion of the genome. An efficient method to reveal the portion of BAC clones derived from the gene-space has the potential for tremendous cost savings in the context of obtaining the sequences of the vast majority of barley genes. The same approach would potentially accelerate progress in many crop plants and other systems that are not being considered for whole-genome sequencing. Popular overgos might not be appropriate when the objective is to obtain gene-specific results (i.e., to deconvolute the BAC-gene relationships). However, when the objective is maximize the number of gene-bearing BACs found, then it is more cost-effective to use popular overgo rather than unique overgos. In addition to this, one cannot hope to design unique overgos for all the unigenes because, for example, of the presence of gene families. Users must also be careful in using popular overgos in pooling strategies, since they might results in too many positives.

The computational problem arising from the selection of popular oligos is an instance of a more general class of problems, called pattern discovery. Several pattern discovery algorithms have been proposed in the literature and implemented in software tools. A few examples are MEME [18], CONSENSUS [19], GIBBSSAMPLER [20,21] and VERBUMCULUS [22] among others. Although some of these tools are able to give very accurate results on datasets in the order of a few tens of kb, they collapse, typically for lack of primary memory, when asked to process very large datasets.

OLIGOSPAWN differs from other probe-finding and pattern discovery software in several important characteristics. The main advantages brought about by this tool are its speed and relatively low memory requirements for datasets in the range of unigenes and total bases that are typical of the entire transcribed sequence dataset from eukaryotic organisms. Both algorithms (for finding unique and popular oligos) have been carefully engineered to achieve satisfactory speeds on ordinary PCs. Although the actual time is highly dependent on the parameters on which the algorithms are run, the execution of each of the algorithms on the barley unigene dataset typically takes just a few hours on a regular PC.

The main algorithmic ideas behind the design of OLIGOSPAWN were reported in previous publications [23,24]. In this paper we report on the release of OLIGOSPAWN, its usage and limitations. In addition, we present new criteria that were carefully hand-tuned to model the hybridization process. We also discuss in more detail the filtering steps (low complexity, presence of secondary structure, repeat content, etc.) and we report some preliminary biological experiments.

## Implementation

We initially based our oligo design algorithms on the length of 36 bases, following the procedure of Thomas *et al*. [15], where 36-mer "overgo" probes were successfully used to screen several mammalian BAC libraries. For 36-mer overgo probes, two 22-mers create the initial template for DNA synthesis, overlapping in 8 bases, leading to the production of a labeled probe.

Overgo 36-mers probes may anneal to genomic DNA segments and produce a positive hybridization signal even if the 36-mer is not perfectly matched to 36 consecutive bases in the target DNA. In the extreme example, an overgo probe with 35 consecutive perfect matches and a terminal mismatch would hybridize and produce a signal. Similarly, an oligo with 30 consecutive matches and 6 terminal mismatches would also seem likely to hybridize. However an oligo with only 12 consecutive matches and 6 mismatches distributed evenly through the remaining 24 bases would have a very decreased melting temperature and would not produce a signal under standard hybridization and washing conditions. DNA sequencing primers generally are in the range of only 17–22 nucleotides, and a popular microarray format is based on 25-mers. These lengths are sufficient for annealing at moderate temperatures, yet not so long that non-perfect-matches are an overriding issue. Considerations of non-perfectly-matched oligos have recently been described in the context of microarrays [25].

### Unique oligos

In order to increase the likelihood that a 36-mer is unique in the operational sense of hybridizing to only one gene in the genome, we allow no more than 15 consecutive perfect matches, and we place a further requirement on the density of mismatches throughout the remainder of the 36-mer. The unique oligo problem is to identify 36-mers in the unigenes collection such that each 36-mer occurs exactly in one unigene and does not occur exactly or approximately in any other unigene. More specifically, based on the considerations above, we define a 36-mer *p *that occurs in a unigene sequence *s *to be *unique *if all the following conditions are satisfied

• for any 16-mer *x *that occurs exactly in *p*, and any 16-mer *y *that does not occur exactly in *s*, *H*(*x*, *y*) > 0,

• for any 20-mer *x *that occurs exactly in *p*, and any 20-mer *y *that does not occur exactly in *s*, *H*(*x*, *y*) > 1,

• for any 24-mer *x *that occurs exactly in *p*, and any 24-mer *y *that does not occur exactly in *s*, *H*(*x*, *y*) > 2,

• for any 30-mer *x *that occurs exactly in *p*, and any 30-mer *y *that does not occur exactly in *s*, *H*(*x*, *y*) > 3,

• for any 36-mer *x *that occurs exactly in *p*, and any 36-mer y that does not occur exactly in *s*, *H*(*x*, *y*) > 4,

where *H*(*x*, *y*) denotes the the number of mismatches (Hamming distance) between two sequences *x *and *y *of equal length.

Our strategy to identify unique oligos first eliminates all the 36-mers that cannot be unique. The algorithm is based on the following observation. Assume that we have two oligos of size *l*, which disagree in at most *d *positions, that is, there are at most *d *mismatches. Then, they have to share a string of size *l*/[*d*/2 + 1] that contains at most one mismatch. We call substrings of this size the *seeds*. Using this idea, we designed an efficient two-phase algorithm. In the first phase, we cluster all the possible seeds from the unigenes into groups such that within each group, a seed has no more than one mismatch with the other seeds. Then, we extend the flanking regions of a seed, and check whether the extended 36-mer violates any of the above conditions. If so, the extended 36-mer is not unique. Observe that we only need to compare 36-mers within a group because any two 36-mers extended from seeds in different groups are different enough that the conditions above would be immediately satisfied.

### Popular oligos

The popular oligo problem is the problem of finding all the 36-mers that appear (permitting some mismatches outside of an exact core) in a sufficiently large number of unigene sequences. More precisely, we say that a 36-mer *x matches *another 36-mer *y*, if the following condition is true

• *x *and *y *share a consecutive perfect match of 20 nucleotides, called a *core*, and

• either one of the following two conditions is satisfied

- *H*(*x*, *y*) < 3, or

- *H*(*x*, *y*) = 3, and

* for any pair *x'*, *y' *of 25-mers obtained by extending the core, *H*(*x'*, *y'*) < 2, and

* for any pair *x"*, *y" *of 30-mers obtained by extending the core, *H*(*x"*, *y"*) < 3.

**Figure 2:**
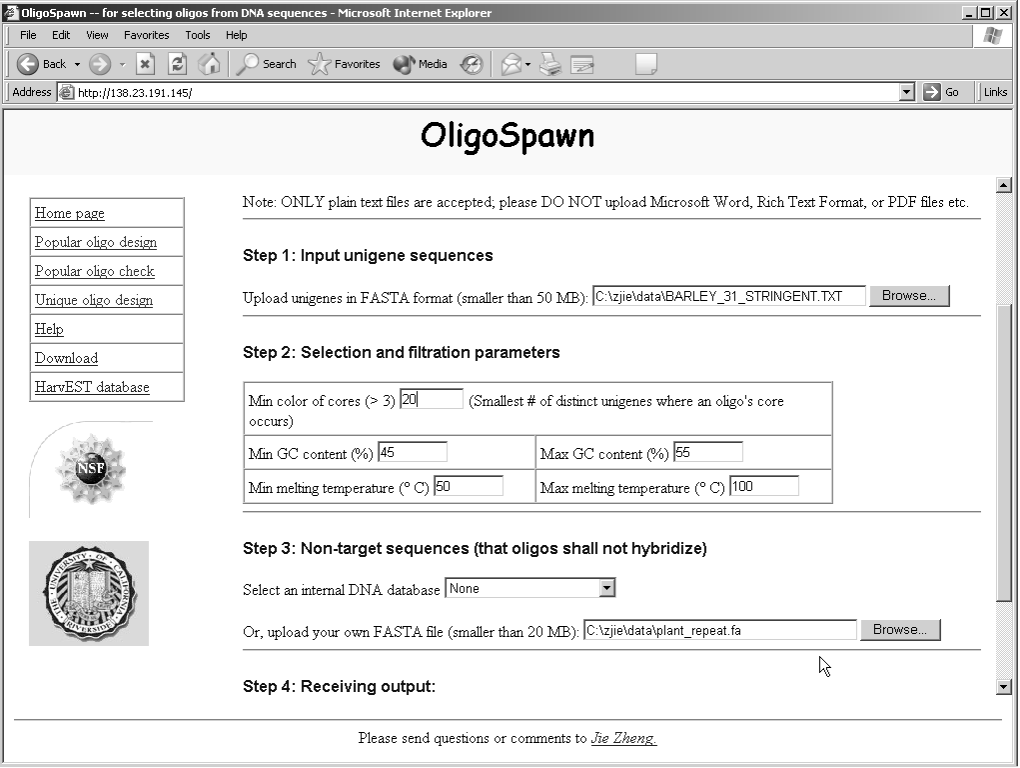
Screenshot of the web interface of OLIGOSPAWN for the popular oligo design.

Since popular oligos are not required to appear exactly in the unigene sequences, it would be too computationally expensive to find them by exhaustive enumeration. However, we can reduce the search space using the same idea as in the algorithm for unique oligos, except that here the role of seeds is played by the cores. First, we determine the popularity of the cores (20-mers) in the unigene dataset. A critical parameter here is the *core threshold T*_*c*_, which is the minimum number of unigenes in which the core should appear exactly to be declared popular (see corresponding parameter in Figure 2). Second, each popular core is extended to a 36-mer. Then, each group of 36-mer is hierarchically clustered. Based on the clustering tree, we compute the common oligos shared by the 36-mers by performing set intersection. These common oligos become candidate popular oligos.

**Figure 1:**
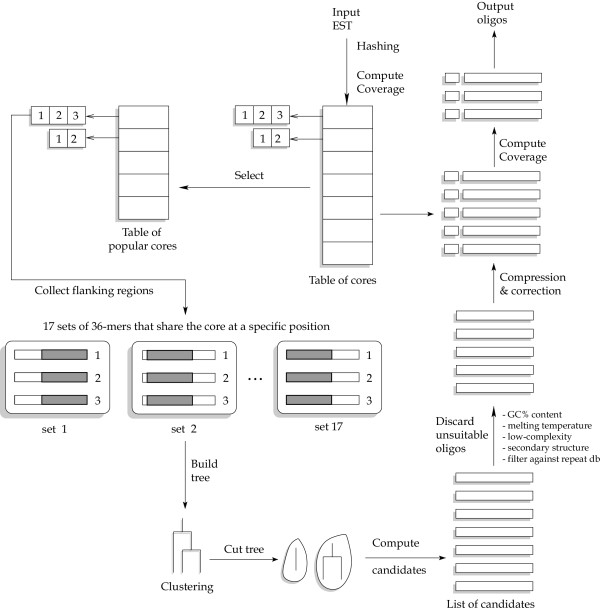
An overview of the algorithm for selecting popular oligos. The length of the oligos is assumed to be 36 bases, and the length of the cores is assumed to be 20 bases. Reproduced with permission from [24].

Both algorithms combine heuristics and well-established algorithmic and data structuring techniques such as hashing, approximate string matching, and clustering. A more detailed explanation can be found in [23,24]. An outline of the popular oligo algorithm is illustrated in Figure 1.

### Filtering unsuitable candidates

For both unique and popular oligos, we apply a filtering phase to discard unsuitable oligos based on GC content, melting temperatures, self-annealing of 36-mers, low-complexity, and the presence of repetitive regions. All these parameters can be adjusted by the user (see Figure 2). The melting temperature *T*_*m *_is calculated using the formula in [26] as implemented in PRIMER3 [11]. Self-annealing of oligos is determined by performing an end-free sequence alignment between the 22-mer prefix and the reverse complement of the 22-mer suffix of an oligo. An oligo is discarded if the alignment score is higher than a predetermined threshold. We use the program DUST [27] to determine low-complexity regions in oligos. Finally, OLIGOSPAWN filters out those popular oligos that have significant matches against repeat database, e.g., Triticeae Repeat Sequence Database (TREP [28]) in the case of barley, or any other repeat database provided by the user.

### Platforms and web interface

**Figure 3:**
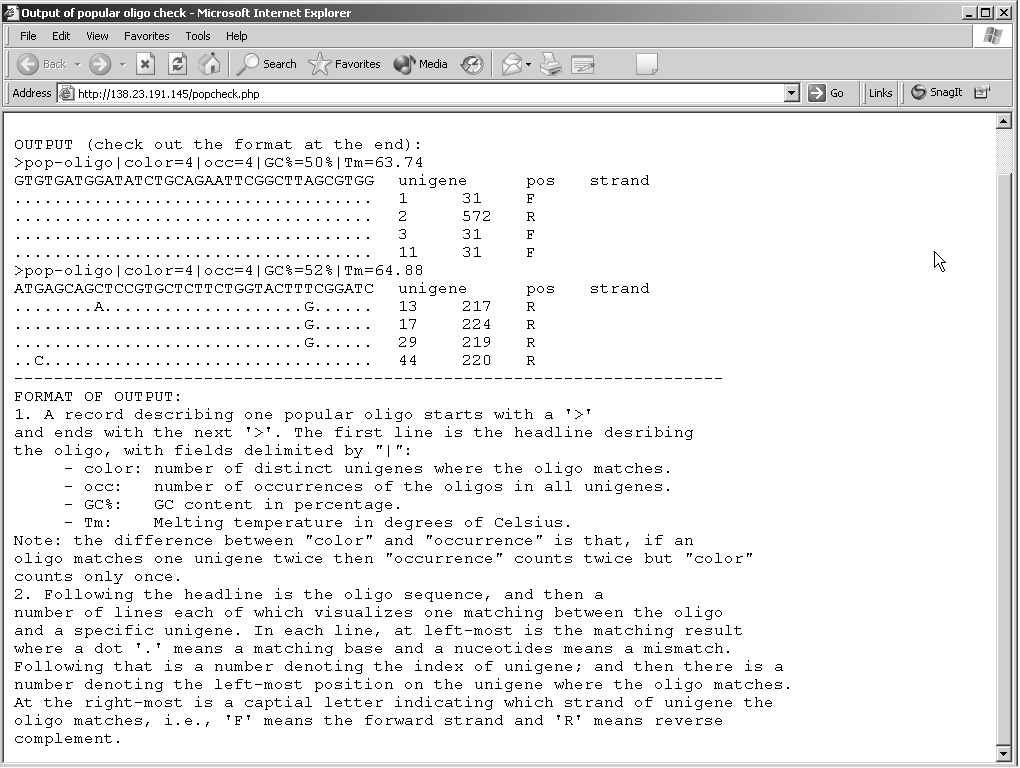
Screenshot of the web interface of OLIGOSPAWN for the oligo check.

The software OLIGOSPAWN was developed using the GNU C++ compiler under the Linux operating system. The executable for Linux/i386 can be downloaded from the OLIGOSPAWN website. The source code is also available from the same website under the GPL licence. Any platform for which GNU C++ is available (Windows and MacOS among others) would be able to compile and run the stand-alone software. The web server is running at [5] and it was developed using PHP [29], which is an open-source scripting language. The web server has been tested with Netscape, Mozilla, Safari, and Internet Explorer. Figure 2 shows a screen shot of the web interface for the popular oligo tool, whereas Figure 3 shows how the output is displayed.

For technical reasons, the web server does not allow inputs larger than 50 megabases. If the dataset is bigger, we suggest the user either to install the software locally or to get in touch with one of the authors of this paper.

### Usage

The large majority of parameters on the website are self-explanatory (GC content, melting temperature, etc.). One of the parameters for the popular oligo, however, deserves a special discussion. The parameter is the threshold *T*_*c *_on the popularity of the cores. If one sets this parameter to a value *v*, he should expect each oligo produced by the tool to hit *v *or more unigenes of the dataset. Although this property is not guaranteed, it should help the intuition in setting *T*_*c*_.

The ideal output of the popular oligo tool is a set of oligos of the minimum cardinality that maximizes the number of unigenes hit by at least one oligo. As reported in [23,24] decreasing the parameter *T*_*c *_has the effect of increasing both the number of oligos in the pool and the number of covered unigenes. However, by doing so the running time grows considerably. More importantly, the ratio of covered unigenes to oligos (*coverage ratio*) decreases as the threshold decreases. For a unigene set containing a few tens of thousands of unigenes we suggest that users start with a value of *T*_*c *_around 20, and then progressively decrease *T*_*c *_until the desired coverage is attained. If *T*_*c *_is low (4 or lower), the running time could be in the order of a few tens of hours on a regular PC for a large unigene datasets (i.e., in the order of a few tens of megabases). Since the other parameters (GC content, melting temperature, etc.) control the selection of the candidates, the stricter is their range, the faster the software will run. If the range is too small, it is possible that no oligo will be reported.

Users who choose to install the software locally will find a README file in the archive explaining the compilation, installation and usage procedures. The two programs can be invoked as follows

pop0ligo.exe unigene.fasta [options]

uniq0ligo.exe unigene.fasta [options]

The options for the two Linux executables are reported in Table 1. For example

**Table 1:** Options for the pop0ligo.exe and uniq0ligo.exe executables

	*flag*	*description*	*default*
pop0ligo.exe	-c <cores color>	minimuin number of unigenes in which a overgo's core must occur *T*_*c *_(must be ≥ 2)	5
	-g <GC%>	minimum GC%	45%
	-G <GC%>	maximum GC%	55%
	-m <temperature>	minimum melting temperature	0°C
	-M <temperature>	maximum melting temperature	100°C
	-r <file.fasta>	fasta file containing the repeat database	

uniqOligo.exe	-g <GC%>	minimum GC%	45%
	-G <GC%>	maximum GC%	55%
	-m <temperature>	minimum melting temperature	0°C
	-M <temperature>	maximum melting temperature	100°C
	-r <file.fasta>	fasta file containing the repeat database	
	-t <number>	number of overgos selected for each unigene	1

$ pop0ligo.exe unigene.fasta -c 5 -g 45 -G 55 -m 50 -M 80 -r trep.fasta

will compute the popular oligos for the unigenes contained in the file unigene.fasta with *T*_*c*_*= *5, GC content between 45% and 55%, and melting temperature between 50°C and 80°C. The program will discard any oligo that matches a sequence in trep.fasta. The executable uniq0ligo.exe has a special option -t that allows the user to choose how many unique oligos to report for each unigene. If several oligo candidates are available for one unigene the program will report the requested number spread evenly across that unigene. The same parameters in Table 1 can be found on the web server.

## Results and Discussion

In order to test and evaluate OLIGOSPAWN, oligos were designed from the unigene set assembly # 32 of HarvEST:Barley [4].

Popular 36-mer oligos were generated by an older version of the software OLIGOSPAWN with threshold *T*_*c *_= 4, GC content in the range 45–56%. Since the older version of OLIGOSPAWN did not yet offer filtering against repeat databases this process was supplemented by some manual actions, as follows. Oligos matching repetitive DNA and rRNA were filtered out with BLAST searches (BLASTn) against TREP and the TIGR *Gramineae *repeat databases (*Hordeum*, *Oryza*, *Sorghum*, *Triticum*, *Zea*) [30,31]. Following this search, 36-mers with 26 or more consecutive matches to repetitive sequences were discarded. Out of 698 initially proposed popular oligos, a total of 25 were discarded by this method. All these filtering step are now included in OLIGOSPAWN (in particular BLAST is not required to run OLIGOSPAWN).

The popular 36-mers were also "blasted" (by BLASTx) against the SwissProt [32] and NR protein databases for annotation purposes. The 36-mers with nine of twelve possible amino acids identical to the subject sequence were chosen for further testing. Out of the initial 698 popular 36-mers analyzed, 134 passed this criterion. Finally, popular oligos classified as transcription and signal transduction components, a total of 18 out of these 134, were used for probing the Morex barley BAC library [33].

### Overgo hybridization

Overgo labeling and hybridization was done essentially as described by Ross *et al*. [6,7]. Briefly, probes were radioactively labeled individually with ^32^P-dATP and ^32^P-dCTP. For background detection, a 36-mer representing the *Escherichia coli *genome was also labeled [7]. Hybridization using a mixture of all 19 probes was then performed on high-density filters of the 6.3× Morex barley BAC library [33], followed by washing and exposure to autoradiography film [6]. An average of 140 BAG clones per filter (17 filters) were scored as positive, yielding a total of about 2,400 positive BAC clones from only 18 popular overgos. Screening with 18 unique overgos would be expected to identify only about 113 total clones (17 × 6.3).

Therefore, the 18 popular oligos described above netted about 22 times as many positive clones as would unique oligos. Results with other sets of popular oligos not described in this manuscript have given comparable results. Therefore, we conclude that the popular oligo algorithm provides a substantial gain of efficiency in probing BAC genomics libraries for gene-containing clones.

The number of positive BAC clones identified with various pools sizes of unique oligos has consistently been in the range of 6 to 8 BACs per unique oligo. For example, pools of 192 unique oligos repeatedly provide about 1,200 to 1,600 positive BAC addresses. Furthermore, checking the sequences of unique oligos with BLAST has consistently provided assurance that our unique oligo algorithm indeed is as selective as it is intended to be.

## Conclusion

The development of OLIGOSPAWN spanned a period of more than two years for conception, design, optimization, tuning, and several cycles of changes in the criteria used to model the hybridization of short oligos. Before OLIGOSPAWN the problem of finding popular oligos from typically large unigene datasets, as exist for barley and several other organisms, could not be solved efficiently using standard desktop computers. Furthermore, most of the commonly used pattern discovery algorithms are not scalable to this level and therefore could not solve this problem using any computer configuration.

Although OLIGOSPAWN uses several heuristics to speed up the computation and therefore cannot guarantee the optimality of the probe it produces, the experiments on the screening of the BAC clone library for barley have demonstrated its effectiveness.

One major limitation of OLIGOSPAWN is that the length of the oligos is currently fixed at 36 bases. It is straightforward to extend the algorithms to accommodate oligo lengths that are not too far from 36 bases, and we are currently working on extending the software to allow the user to specify the oligo length (from a reasonable range).

## Availability and requirement

**Project name: **OLIGOSPAWN

**Project homepage: **

**Operating system: **Linux

**Programming languages: **C++, PHP

**Licence: **GNU GPL

**Any restriction to use by non-academics: **no

## Authors' contributions

JS and TC designed the specification of the overgos and the criteria to model the hybridization. JZ, TJ and SL designed the two algorithms. JZ wrote and tested the software OLIGOSPAWN. JS and KM performed the overgo labeling and hybridization experiments. All authors read and approved the final manuscript.
